# Transcriptomic landscape of early age onset of colorectal cancer identifies novel genes and pathways in Indian CRC patients

**DOI:** 10.1038/s41598-021-91154-x

**Published:** 2021-06-03

**Authors:** Manish Pratap Singh, Sandhya Rai, Nand K. Singh, Sameer Srivastava

**Affiliations:** Department of Biotechnology, Motilal Nehru National Institute of Technology Allahabad, Prayagraj, 211004 India

**Keywords:** Cancer, Molecular biology

## Abstract

Past decades of the current millennium have witnessed an unprecedented rise in Early age Onset of Colo Rectal Cancer (EOCRC) cases in India as well as across the globe. Unfortunately, EOCRCs are diagnosed at a more advanced stage of cancer. Moreover, the aetiology of EOCRC is not fully explored and still remains obscure. This study is aimed towards the identification of genes and pathways implicated in the EOCRC. In the present study, we performed high throughput RNA sequencing of colorectal tumor tissues for four EOCRC (median age 43.5 years) samples with adjacent mucosa and performed subsequent bioinformatics analysis to identify novel deregulated pathways and genes. Our integrated analysis identifies 17 hub genes (*INSR, TNS1, IL1RAP, CD22, FCRLA, CXCL3, HGF, MS4A1, CD79B, CXCR2, IL1A, PTPN11, IRS1, IL1B, MET, TCL1A*, and *IL1R1*). Pathway analysis of identified genes revealed that they were involved in the MAPK signaling pathway, hematopoietic cell lineage, cytokine–cytokine receptor pathway and PI3K-Akt signaling pathway. Survival and stage plot analysis identified four genes *CXCL3, IL1B, MET* and *TNS1* genes (*p* = 0.015, 0.038, 0.049 and 0.011 respectively), significantly associated with overall survival. Further, differential expression of *TNS1* and *MET* were confirmed on the validation cohort of the 5 EOCRCs (median age < 50 years and sporadic origin). This is the first approach to find early age onset biomarkers in Indian CRC patients. Among these *TNS1* and *MET* are novel for EOCRC and may serve as potential biomarkers and novel therapeutic targets in future.

## Introduction

In terms of incidence and cancer related deaths, colorectal cancer (CRC) is one of the most common cancers in the world. As per recent reports, it was projected that there would be over 1.8 million new cases and 881,000 deaths of CRC in 2018. Rate of incidence of CRC are about threefold higher in lower or middle-income countries than in developed countries^1^. In 2019, new cancer cases were estimated to be 1,762,450 and deaths were estimated to be 606,880 in the USA alone^2^.

There has been a decreasing trend in CRC incidence and mortality rate in the past two decades, however, Early age Onset of Colo Rectal Cancer (EOCRC) incidence rates are progressively increasing worldwide. This has initiated a new point of discussion for age dependent average risk screening for CRC in future^3,4^. Initially standard age for average risk screening was 50 years, but the increasing burden of early age onset has forced to revise the standard age for risk screening to 45 years^5^.

In India, population-based studies on CRC are limited to government cancer registry programs and systematic or community-based testing of CRC is not performed^6^. The incidence of CRC occurrence is gradually increasing year by year. Recent statistical data suggests that it became the fourth major cause of adult death in rural areas, whereas the second most prime cause of death in urban areas^7^. Mathew et al., 2019, reported that in India, overall CRC incidence are gradually increasing (~ 18% in 2004–05 to ~ 27% in 2012–14), which is contrary to western countries^8^. However, India follows a similar pattern of increasing rate of incidence for EOCRC. It is imperative to mention that EOCRC incidence rate has increased about 30% in a decade in India (2.7% in 2004–05 to 3.5% in 2012–2014)^8^.

Early age CRC, pathologically has poor histological differentiation, higher content of signet ring cell, tends to be left sided and gets diagnosed at an advanced stage. Most of the early age CRC patients are affected by heavy mutational load and about 20% have familial origin^9^.

Recent transcriptome based studies have provided a better interpretation of pathways and mechanisms involved in the onset and progression of early age colorectal carcinomas. Myriads of genetic changes are involved in the adenoma to carcinoma progression and various molecular signatures have been identified through significant methodological and technological advances. For example, *EZH2* and *COX2* were used as a biomarker in early diagnosis as both were upregulated in adenoma and carcinoma^10^. Whole genome profiling of early age CRC patients have identified high mutation load in MAPK signaling pathway, while late-onset were deregulated in PI3K-AKT pathway genes^11^. However, amidst recent reports, it would not be an overstatement that aetiology and biomarkers for timely diagnosis of EOCRC are still obscure and need more attention.

In this study, we performed high throughput RNA sequencing of early age CRCs (with adjacent mucosa) and its subsequent bioinformatics analysis to identify novel deregulated pathways and genes in EOCRC. Further, we validated these DEGs through quantitative real-time PCR. The analysis outcome provides new insight on distinctive features of EOCRC and the discovery of novel biomarkers that may have potential in diagnostic and therapeutic settings. As per our knowledge, this would be the first attempt to perform transcriptomic analysis of early age onset of CRC (EOCRC) patients in India.

## Materials and methods

### Patient samples and demographic details

Patient samples have been scrutinized following the set criteria. For this study, our aim was to search DEGs unique to EOCRCs. A total of 70 tumors and 40 matched normal colonic mucosa samples were collected from RGCIRC (Rajiv Gandhi Cancer Institute and Research Centre), India, during 2016 to 2019. It was ensured that the tumor was sporadic and the patient has not received any chemotherapy or radiotherapy before surgery. The total oncogenic area of cancerous cells was ensured to not less than 80% by pathologist and MSI status of each sample was also evaluated. Informed consent was obtained from each patient. The study was approved by the Institute Ethics Committee, Motilal Nehru National Institute of Technology Allahabad (Ref. No. IEC17-18/027). Subsequently, considering desired clinical parameters (Early age onset (below 50 years), no chemotherapy & radiotherapy received and sporadic origin), we screened four tumor samples. Demographic details of the discovery cohort (4 tumor samples and 1 adjacent mucosa of sample No. 2) are provided in Table 1. Care was taken in selecting these tumor samples as they were cases of early onset (with mean age: 43.5 years) and no family history.

**Table 1:** Clinicopathological features of four tumor samples (discovery cohort).

S no	Gender	Age	Location	Histological type	Differentiation	Stage
1	M	36	Ascending colon	Mucinous adenocarcinoma	Poorly	IIIB
2*	F	45	Ascending colon	Medullary carcinoma	Poorly	IIA
3	F	46	Ascending Colon	Adenocarcinoma	Moderate	IIIB
4	M	48	Transvers colon	Adenocarcinoma	Poorly	IIB

*Sample 5 was paired normal of tumor sample no. 2.

### RNA extraction and library preparation

Total RNA was extracted from tumor tissues using Monarch Total RNA Miniprep Kit (NEB) and RNA concentration and quality was estimated using Qubit 3.0 Fluorometer and Tape station. Samples having good RIN (5.5–7.5) qualified for cDNA conversion and paired end library preparation and sequenced using Illumina NextSeq 500 system. It generates 150 bp paired end raw reads and these were quality checked for low quality bases and adapter sequences (Processing of raw reads). Quality check was done using Perl scripts followed by RNA-Seq reads alignment with reference human genome using TopHat2 and finally, differential expression analysis of reads was performed by Cufflinks generating up/down/ neutral DEGs for further analysis.

### Quantitative real time PCR analysis (QRT-PCR)

We performed QRT-PCR analysis on two sets of samples. To confirm our NGS data analysis, QRT-PCR of top 7 up regulated and top 7 down regulated common genes (*p* < 0.05) were performed on discovery cohort (4 samples) along with their paired adjacent mucosa. Gene expression analysis by QRT-PCR was also performed for the most significant hub genes (identified by our comprehensive functional analysis) on the discovery cohort (samples sent for NGS analysis) for replicating the gene expression results obtained from our NGS data analysis and on the EOCRC cohort (validation cohort: 5 samples) with their paired adjacent mucosa to validate the expression status. Briefly, Total RNA was isolated from tumor samples using HiPurA Total RNA Miniprep Purification Kit (Himedia) and extracted RNAs were checked for quality, integrity and quantity using agarose gel and spectrophotometer (NanoDrop 2000, Thermo Scientific) and stored at − 80 °C until further use. Total RNA (1 µg) was used to prepare cDNA using iScript cDNA conversion kit (BioRad) in a 20 µl reaction according to the manufacturer’s instructions. Prepared cDNA was diluted 10 times and 1 µl each was used in all QRT-PCR reactions. QRT-PCR was performed using SYBR green chemistry. GAPDH was used as internal control for relative expression calculations (2^–∆∆CT^). Primers for all the selected genes used in the analysis are given in Supplementary Table 3. All the QRT-PCR experiments were performed in triplicates.

### Gene ontology and pathway analysis

Gene ontology (GO) analysis was performed to find out the functional role of DEGs common among all data sets using iDEP 9.0^12^, SHINNY GO^13^, and MONA GO^14^ online servers that are based on DAVID Gene enrichment analysis tool. Shortlisted DEGs were subsequently processed for enrichment analysis including biological process (BP), molecular function (MF) and cellular components (CC). Heat map of top upregulated and downregulated DEGs was constructed on iDEP 9.0 and a cut off FDR (false discovery rate) < 0.05 was considered to be statistically significant. KEGG pathway analysis was performed on SHINNY GO enrichment analysis server (part of iDEP project)^15^. It creates a network of highly significant genes in biological pathways. MONA GO was used to create a cumulative circular chord diagram that comprehensively represents the number of genes individually and overlapping in all gene enrichment processes (BP, MF, and CC). The processes were considered as statistically significant at corrected *p* < 0.05.

### Establishment of PPI network and Identification of hub genes

To establish and visualize the predicted association with the proteins, protein–protein interaction (PPI) network was constructed on STRING database^16^. The analysis was performed using default parameters. The PPI network analysis file containing data for interacting proteins, edges and nodes was imported to Cytoscape^17^ to visualize their interaction and create PPI network. Again, these DEGs were processed in iDEP 9.0 to create clusters of highly connected genes within the different networks. Cytohubba plugin was used to identify the hub genes among the screened DEGs. This plugin was supported on Cytoscape platform. Cytohubba analyses hub genes on two or more than two algorithms DMNC and MCC. Both the algorithms provide different hub genes in a similar set of genes.

### Survival analysis of hub genes on TCGA and cancer related pathways in KEGG database

To identify the most significant genes and pathways, survival and stage plot analysis of selected hub genes were performed on GEPIA online tool using TCGA data of colorectal adenocarcinoma (COAD)^18^. Further, we performed the pathway analysis for candidate hub genes on the STRING database to explore the enriched KEGG pathway and interacting clusters of hub genes.

### Ethical statement

All the experiment protocol for involving human data was in accordance to guidelines of national/international/institutional or Declaration of Helsinki in the manuscript**.**

## Results

### NGS data collection and analysis

RNA-Seq data of four tumor samples (Mean age: 43.5 years) with adjacent mucosa were used for the NGS based data analysis. All the paired end raw reads generated in each sample were scrutinized for quality parameters and low-quality reads were removed from the raw reads using Perl script. NGS generated overall 39.6 GB raw reads. After quality check, 37.3 GB clean reads were processed. The processed and raw reads have been reported in Supplementary Table 1. After all quality checks, the clean processed reads were mapped and aligned with *Homo sapiens* (reference genome) genome using TopHat 2.0 (built on the ultrafast short read mapping program Bowtie). In all five samples (including adjacent mucosa of sample no. 2), the average mapping ratio calculated was 81.96%. The Supplementary Fig. 1 represents mapping frequency for all samples with multiple % read alignment.

### Differential gene expression analysis

**Figure 1:**
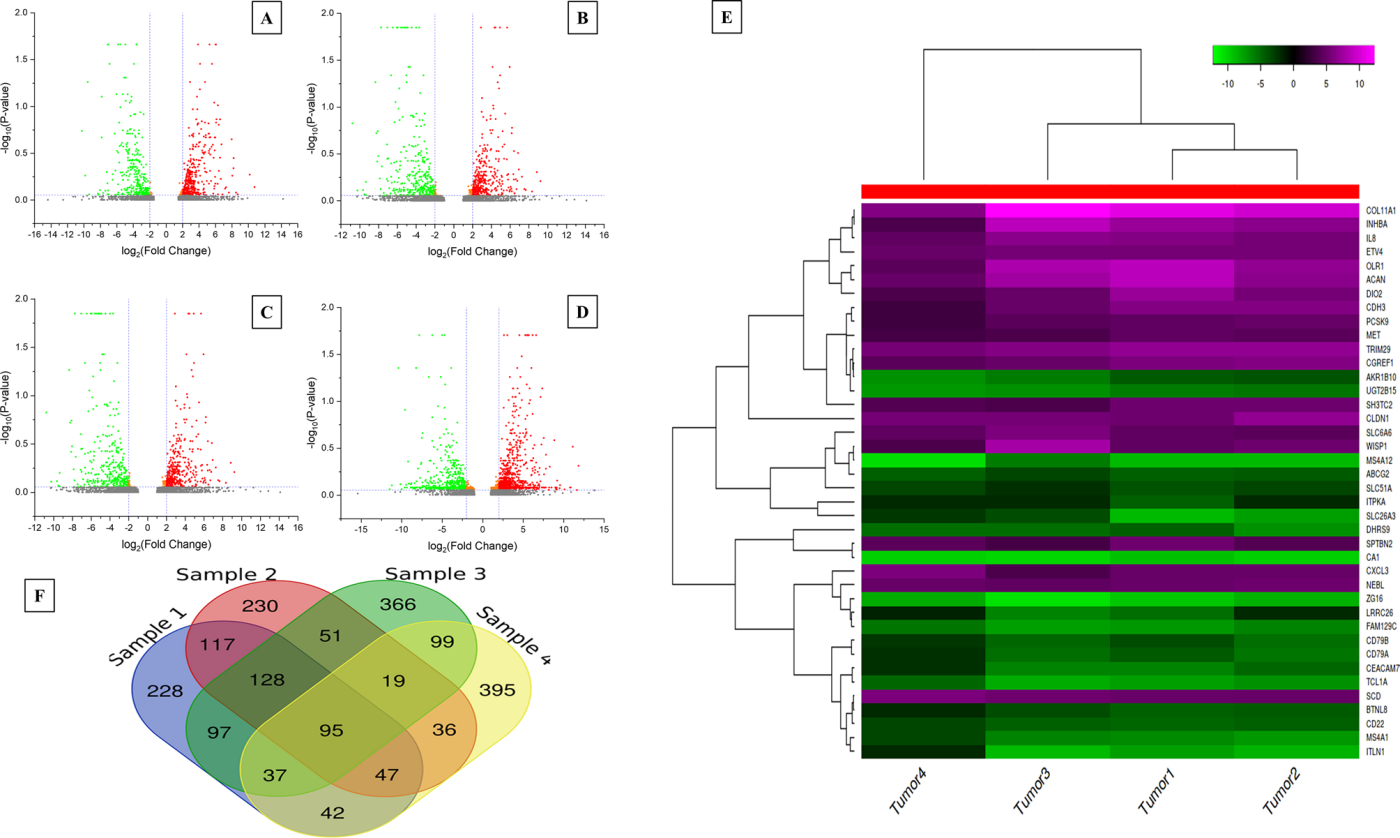
Identification of DEGs in tumor samples. (**A**), (**B**), (**C**), (**D**) represents Volcano plot of all expressed genes in each pairwise comparison in tumors 1, 2, 3 and 4, respectively. The *x*-axis shows the log 2 (fold change) and the *y*-axis shows the – log 10 (1 − *P*). Each dot is a differentially expressed gene (DEG). Red dots denote upregulated DEGs and green dots are downregulated DEGs. (**E**) Expression heat map of top 20 down and upregulated DEGs. (**F**) Venn analysis of common DEGs among 4 tumor samples for log 2 (fold change) ± 2.

TopHat 2.0 and cufflinks pipeline was used for RNA-Seq analysis, to assemble transcripts, measure their frequency and check differential expression. We calculated the total number of genes upregulated and downregulated in all samples. Adjacent mucosa of sample no.2 was taken as normal control for differential gene expression analysis. Four tumor samples were represented as four different data sets against normal control. Initially, we considered log_2_fold change ≥ 1 (*p* < 0.05) for upregulated and ≤ − 1 (*p* < 0.05) for down regulated transcripts. The comparison of transcripts, for differentially expressed genes against control samples, has been represented in Supplementary Table 2. Further, the cutoff criterion for log fold change was increased to log_2_ (≥ 2 and ≤ − 2) fold for comprehensive analysis of the potential DEGs in the data set. Volcano plot for each sample has been displayed in Fig. 1A–D. These were generated having cutoff criteria log_2_fold change ≥ 2 (*p* < 0.05) for up regulated and ≤ − 2 (*p* < 0.05) for down regulated genes. Among all samples that displayed variable number of genes, we further identified common DEGs that were frequently over expressed and under expressed in all tumor samples. Finally, 95 genes were screened that were considered as significant DEGs and out of them 47 genes were upregulated and 48 genes were downregulated (Fig. 1F). The heat map was generated using hierarchical clustering method having cutoff Z score 4 on average linkage and distance was based on correlation. The expression heat map of top 20 up and down regulated DEGs demonstrates important clusters with the possibility of co-expressed DEGs (Fig. 1E). The figure can be grouped into top 14 co-upregulated genes and 14 co-under expressed genes. It was also noteworthy that genes of similar or related pathways were co-overexpressed (Top panel: Fig. 1E) or co-under expressed (Bottom panel: Fig. 1E). Heat map for all common DEGs is displayed in Supplementary Fig. 2.

### Validation of transcriptomic data using QRT-PCR

**Figure 2:**
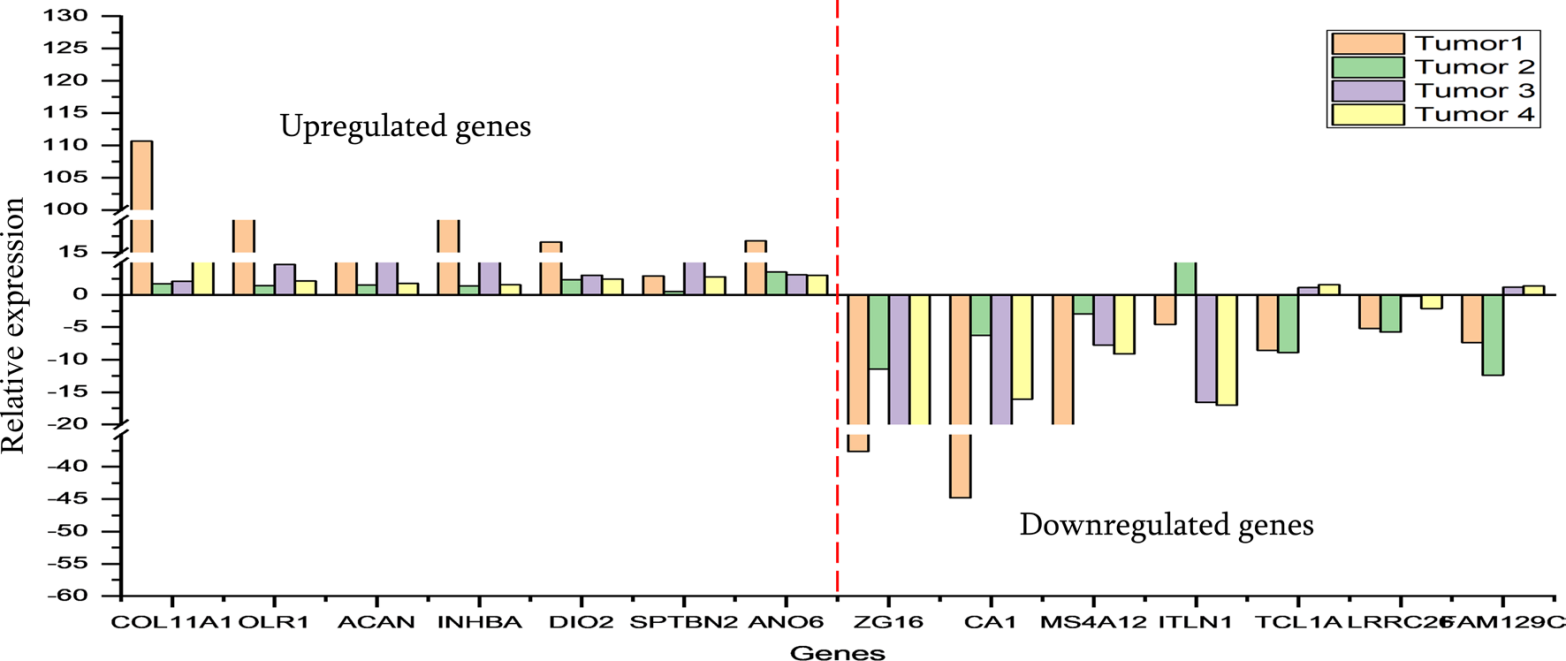
QRT-PCR analysis of upregulated and down regulated DEGs from RNA-Seq data analysis. The left panel of genes represents up regulated DEGs and the right panel of genes represents downregulated DEGs.

In order to validate and confirm our bioinformatics pipeline for DEGs identification, we selected seven up and down regulated genes which were identified to be common in all the four samples. We performed QRT-PCR analysis for these selected set of genes on 4 tumour samples (discovery cohort) along with their adjacent mucosa as control. Similar expression pattern was observed for the above seven up and down regulated candidate genes in all 4 tumor samples supporting our NGS data analysis results. Relative expression analysis confirmed the robustness of these DEGs with few exceptions. Figure 2 demonstrates the distribution of differentially expressed genes among complete dataset.

### Functional analysis of common DEGs in EOCRC

**Figure 3:**
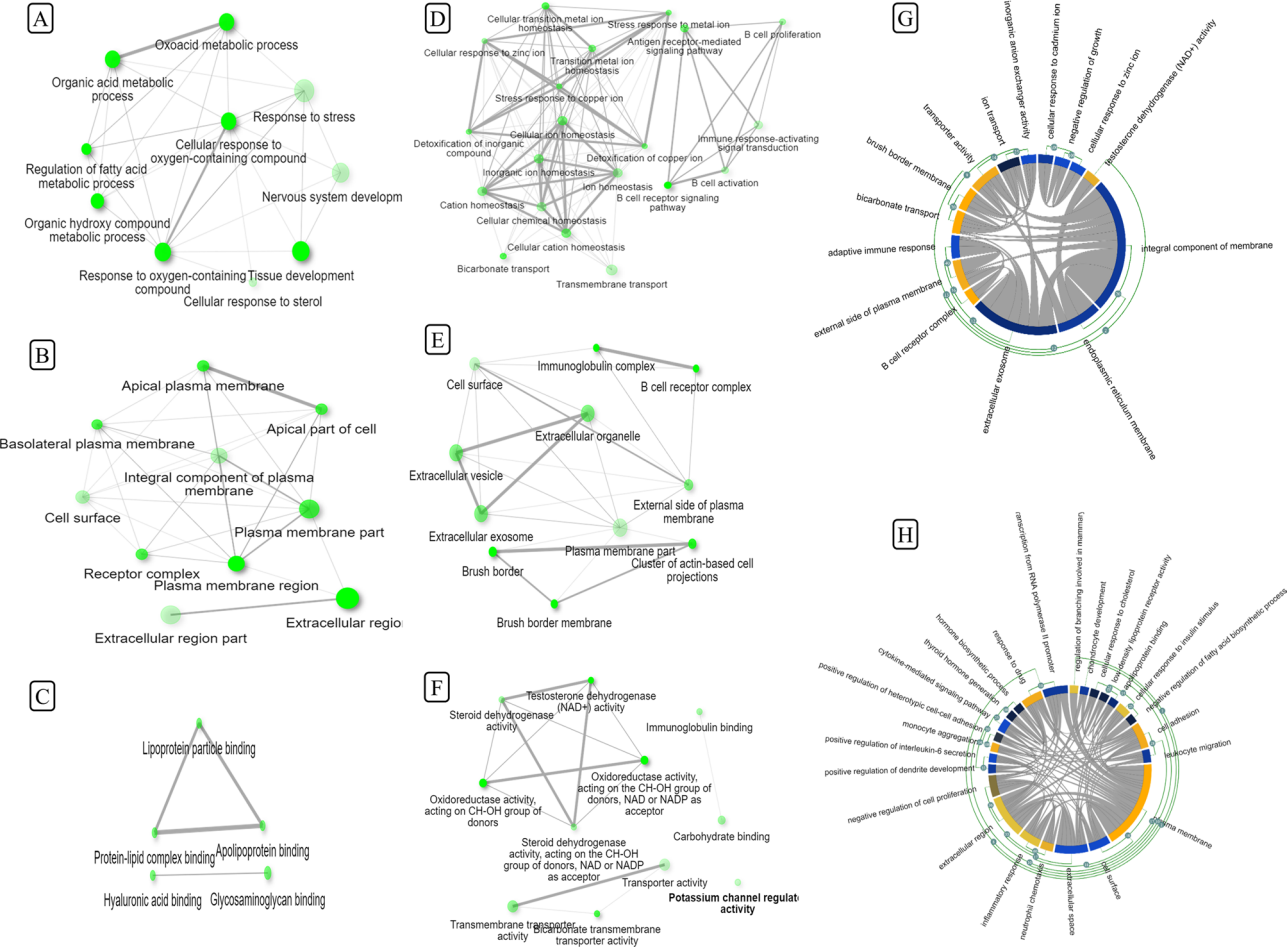
Functional characteristic analysis of DEGs on Shinny GO v0.61 (http://bioinformatics.sdstate.edu/go/). (**A**–**C**) represents the gene ontology enrichment for upregulated DEGs and (**D**–**F**) represents the gene ontology for down regulated DEGs. (**A**) and (**D**) networks are DEGs for biological process (BP), (**B**) and (**E**) are cellular components (CC) and (**D**) & (**F**) networks are for molecular function (MF) gene enrichment analysis. In each network the size of the node denotes no. of genes involved in a particular function and intensity of color denotes the significance of interaction. MONA GO (https://monago.erc.monash.edu/) (**G** and **H**) represent the cumulative biological processes for upregulated and downregulated DEGs, respectively.

Functional role of selected up regulated (47) and downregulated (48) DEGs was analyzed on MONA G, iDEP and SHINNY GO online functional enrichment servers. The analysis was performed for three major categories; BP, MF and CC. The up regulated DEGs analyzed for BP were mainly enriched in cellular processes followed by metabolic processes and biological regulation. Molecular function category contains majorly binding activity genes followed by catalytic and transcriptional regulator activity genes. Cellular component analysis identified that up regulated DEGs were primarily grouped into cell junction, cell function and extracellular region proteins. Protein class analysis revealed that up regulated DEGs were grouped into nucleic acid binding, transcription factors, signaling molecules and transferases proteins. Pathway analysis of up regulated DEGs demonstrated that the majority of genes are involved in Wnt signaling, insulin/IGF, PI3K pathway, TGF beta signaling and angiogenesis related pathways. On the other hand, GO enrichment analysis of down regulated DEGs for BP, MF and CC revealed that most of the DEGs were related to binding and catalytic activity. Cellular process proteins responsible for cell communication, cell signal transduction and cellular response to stimuli are the main components for biological processes. CC group was enriched in extra cellular transporters and binding functions. Pathway analysis revealed that down regulated DEGs were enriched in B cell activation, apoptosis and interleukin signaling pathways. The conclusive pathway analysis revealed that these DEGs are involved in cancer related pathways and biological processes (Fig. 3).

### Establishment of PPI network and module analysis

DEGs were next analyzed by string database and iDEP 9.0 database to create PPI network and module analysis. In the PPI network, 92 nodes and 91 edges were generated including both up regulated and down regulated DEGs. PPI enrichment *p* value (< 1.0e−16) demonstrates that the current network has high interaction probability among themselves and is statistically significant. Module analysis was performed by iDEP and it generated 4 different modules for best grouped proteins based on WGCNA (Weighted Co-expression Network Analysis) co-expression network having a soft threshold of 5 and edge threshold of 0.4 for top 10 genes (Fig. 3). Module 1 genes were rich in activation of immune response, antigen receptor mediated signaling pathway and B cell proliferation related signaling pathway. Module 2 was enriched in cytokine biosynthesis, Interleukin-2 biosynthesis and aminoglycon biosynthetic process. Module 3 genes were primarily enriched in metabolic process related genes such as phosphorus metabolic process, organic hydroxy compound metabolic process and alcohol metabolic process.

### Identification of comprehensive set of Hub genes using PPI network

**Figure 4:**
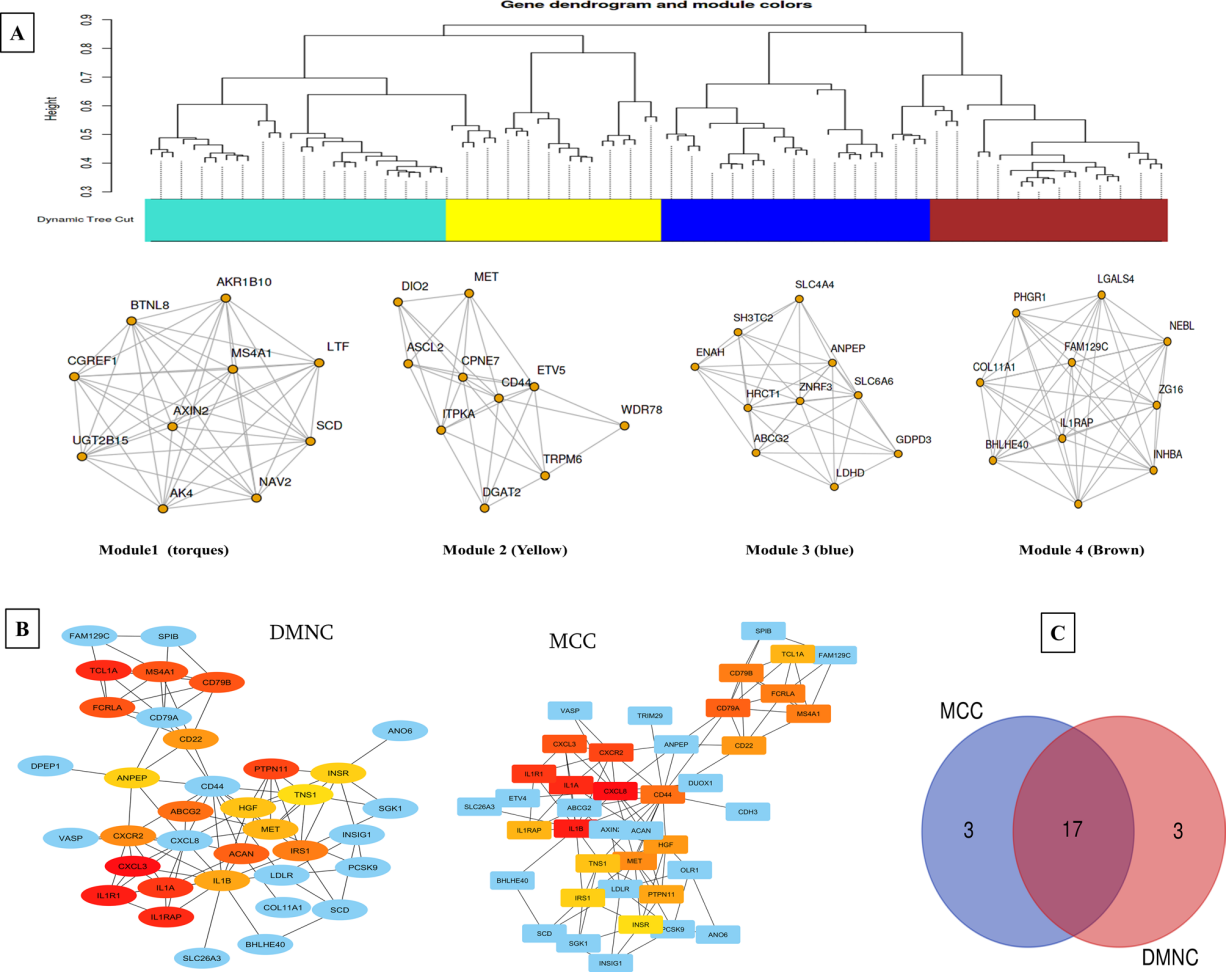
Analysis of PPI interaction and hub genes identification. (**A**) Co-expression networks and sub-modules using WGCNA (Weighted correlation network analysis) of DEGs on iDEP 9.0 (http://bioinformatics.sdstate.edu/idep/) generates modules of the top 10 highly correlated genes. (**B**) Identification of significant hub genes from DEGs using DMNC and MCC algorithm on Cytoscape 3.7.1. Color represents the ranked hub genes on Cytoscape based on the corrected *p* value. (**C**) Venn analysis of both sets of hub genes to find common significant hub genes (http://bioinformatics.psb.ugent.be/webtools/Venn/).

The selected DEGs were analyzed for identifying hub genes using Cytohubba plugin on Cytoscape platform. Top 20 genes were screened based on two algorithms; DMNC and MCC. MCC identified *CXCL8, IL1B, IL1A, IL1R1, CXCR2, CXCL3, CD79A, CD44, MS4A1, FCRLA, CD79B, MET, HGF, CD22, PTPN11, TCL1A, IL1RAP, TNS1, IRS1*, and *INSR* genes while DMNC identified *CXCL3, IL1R1, TCL1A, IL1RAP, IL1A, PTPN11, MS4A1, FCRLA, CD79B, ACAN, ABCG2, IRS1, CXCR2, CD22, IL1B, MET, HGF, ANPEP, INSR, TNS1* genes. Venn analysis of these genes provided a set of 17 common gene list (*INSR, TNS1, IL1RAP, CD22, FCRLA, CXCL3, HGF, MS4A1, CD79B, CXCR2, IL1A, PTPN11, IRS1, IL1B, MET, TCL1A*, and *IL1R1*) and rest six genes in both sets were excluded from further analysis (Fig. 4).

### Key cancer related genes and pathways implicated in EOCRC

**Figure 5:**
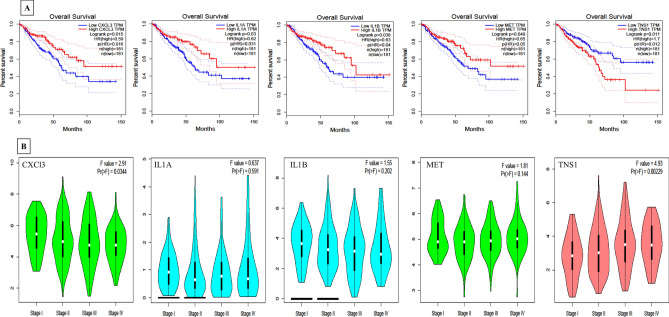
TCGA based validation and survival analysis on GEPIA (http://gepia.cancer-pku.cn/index.html). (**A**) Overall survival analysis of significant hub genes on TCGA using the COAD cohort. (**B**) Gene expression analysis of *TNS1, CXCL3, MET, IL1B* and *IL1A* in all stages (I–IV) of CRC.

**Figure 6:**
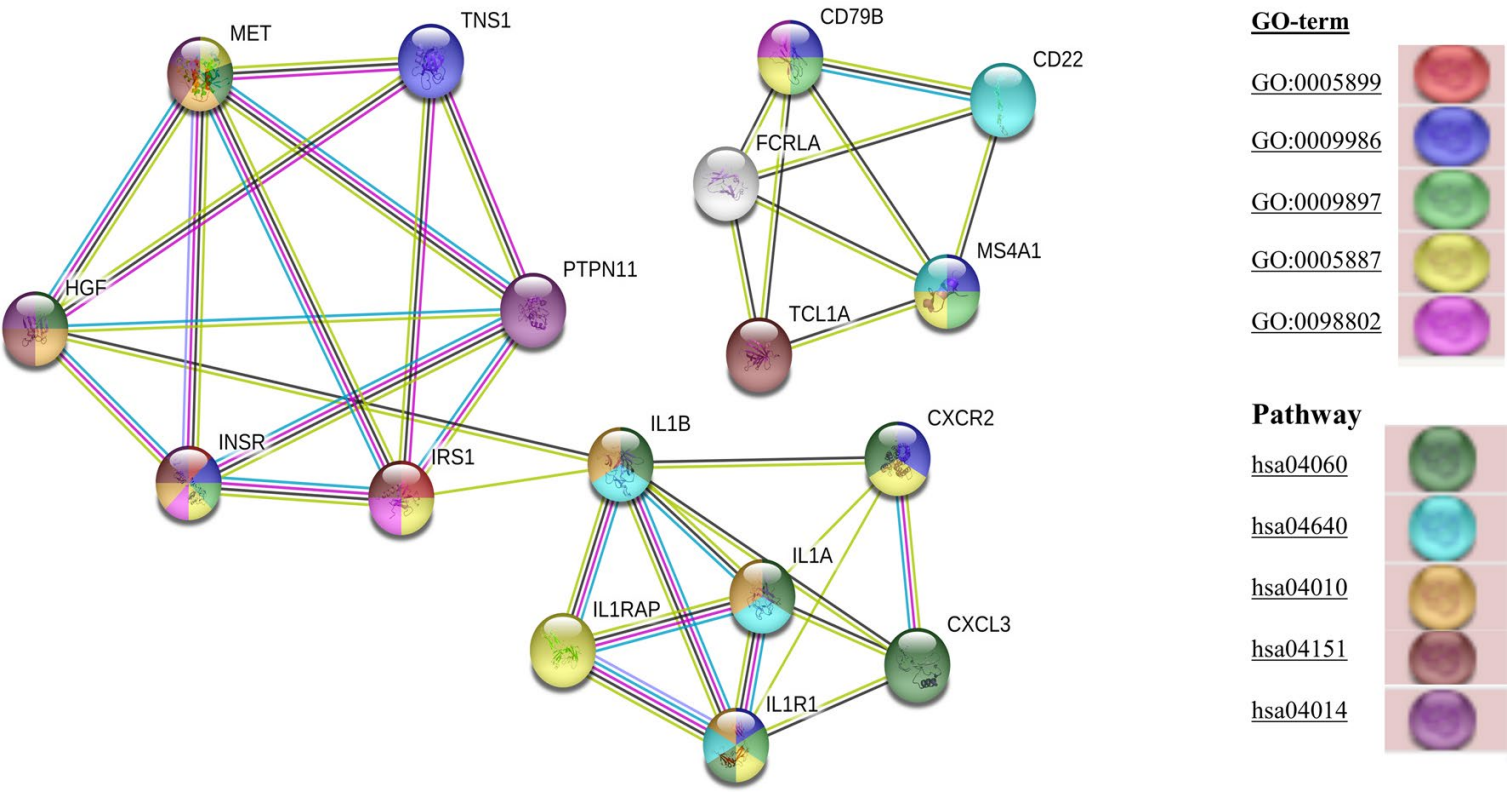
Pathway analysis and cellular processes analysis of Hub genes on string database version 11.0 (https://string-db.org/). Each node is representative of a gene interacting within the clusters. The nodes present in the clusters are filled by different colors respective of the pathway shared by that gene having significant FDR in the KEGG database. The color codes are provided in the right corner and details of respective pathways are provided in Table 2.

The common 17 hub genes were tested on TCGA data set of colorectal adenocarcinomas (COAD) using matched TCGA normal dataset. These genes were significantly and constantly differentially expressed among large data sets. Similar set of expression patterns was followed by our transcriptomic dataset for these 17 genes (Supplementary Fig. 3). These set of genes were further analyzed for survival analysis and expression among different stages of cancer. Survival analysis on GEPIA using colon adenocarcinoma data set screened 5 genes *CXCL3, IL1B, IL1A, MET* and *TNS1* that have significant log rank p value in Kaplan–Meier survival analysis. The graph represents *p* = 0.015, 0.038, 0.03, 0.049 and 0.011 for *CXCL3, IL1B, IL1A, MET* and *TNS1* respectively, suggesting significant association of these genes with overall survival (Fig. 5). Hub genes which qualified in survival analysis were next screened for stage dependent distribution of these DEGs in colorectal cancer in TCGA database. *TNS1* (*p* = 0.00229), *CXCL3* (*p* = 0.034), *MET* (*p* = 0.144), and *IL1B* (*p* = 0.202) were significantly distributed stage-wide with higher expression in tumors. However, *IL1A* (*p* = 0.591) was least associated with stage dependent distribution (fold expression very low and insignificant across all stages) (Fig. 5). These 17 common hub genes were now analyzed for KEGG pathway and cellular process on the STRING database. The colour code for each gene represents the gene involved in the various pathways and cellular processes with significant FDR (Fig. 6). The nodes present in the clusters are filled by different colors respective of the pathway shared by that gene in the KEGG database. *MET* gene was involved in Cytokine–cytokine receptor interaction (FDR 1.23e−07), MAPK signaling pathway (FDR 3.50e−06), PI3K-Akt signaling pathway (FDR 0.00016), Ras signaling pathway (FDR 0.00036) and integral component of plasma membrane (FDR 0.00099). Most of the genes in the cluster were involved in the MAPK signaling pathway, hematopoietic cell lineage, cytokine–cytokine receptor pathway, PI3K-Akt signaling pathway and their corresponding characteristics are displayed in Fig. 6 and pathway they represent are given in Table 2 with corresponding FDR.

**Table 2:** KEGG pathway and Cellular processes analysis of Hub genes implicated in EOCRC progression on STRING database. (A) Cellular process analysis of Hub genes on string database. (B) KEGG pathway analysis of Hub genes on string database.

(A)	GO-term	Description	Count in gene set	False discovery rate
1	GO:0005899	Insulin receptor complex	2 of 2	0.00048
2	GO:0009986	Cell surface	6 of 690	0.00099
3	GO:0009897	External side of plasma membrane	4 of 223	0.00099
4	GO:0005887	Integral component of plasma membrane	8 of 1564	0.00099
5	GO:0098802	Plasma membrane receptor complex	3 of 158	0.0065

(B)	Pathway	Description	Count in gene set	False discovery rate
1	hsa04060	Cytokine–cytokine receptor interaction	7 of 263	1.23e−07
2	hsa04640	Hematopoietic cell lineage	5 of 94	7.12e−07
3	hsa04010	MAPK signaling pathway	6 of 293	3.50e−06
4	hsa04151	PI3K-Akt signaling pathway	5 of 348	0.00016
5	hsa04014	Ras signaling pathway	4 of 228	0.00036

### Validation of TNS1 and MET gene expression in EOCRC

**Figure 7:**
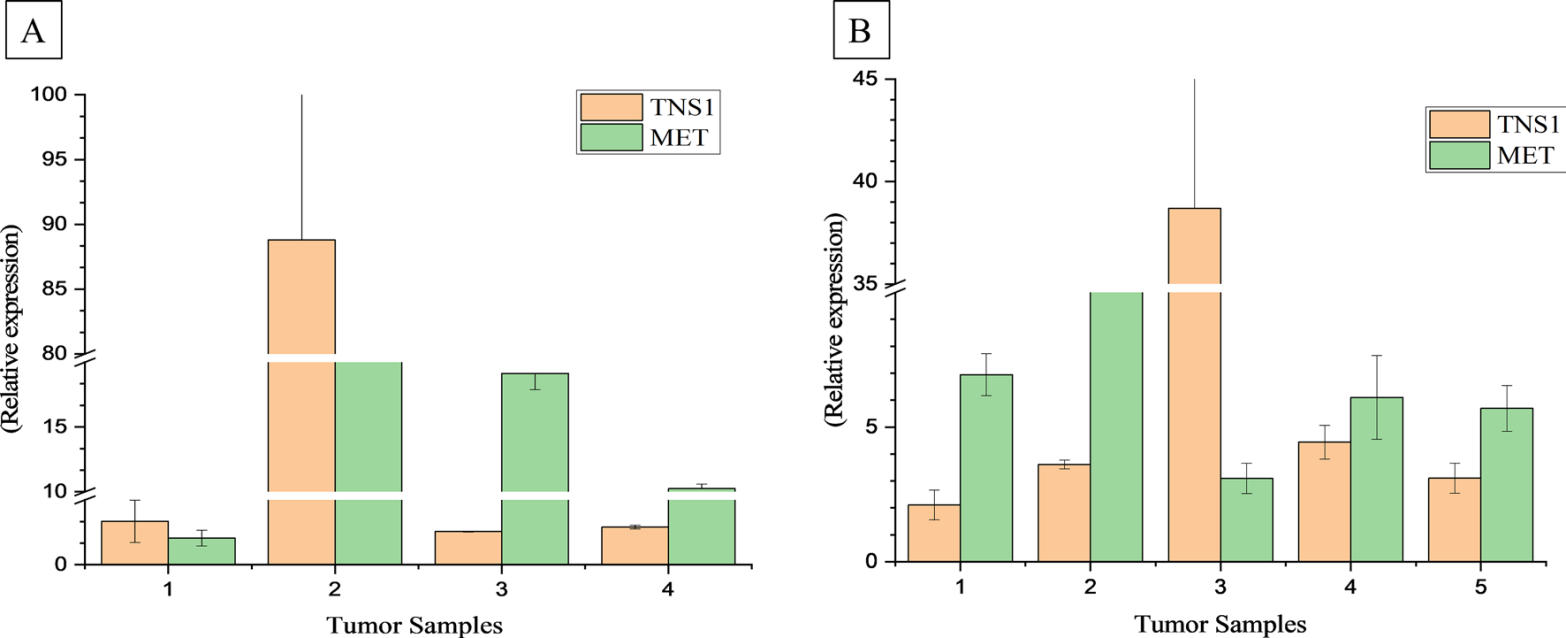
Differential expression analysis of *TNS1* and *MET* gene on EOCRC. (**A**) Represents the relative expression analysis 4 tumor samples of the discovery cohort and (**B**) Represents the relative expression analysis of *TNS1* and *MET* gene on validation cohort.

To support our analysis, gene expression studies were performed for the two significantly upregulated genes i.e. *TNS1* and *MET* in the validation cohort of 5 EOCRC patients. Out of 70 tumor samples, 15 were identified as EOCRC (diagnosed at 50 year or less) patients, and out of them only 9 samples were of sporadic origin (including 4 samples used in the transcriptomics analysis (discovery cohort)). So, validation analysis was performed on the 5 EOCRC samples using QRT-PCR. QRT-PCR analysis demonstrated that *TNS1* and *MET* were significantly up regulated in all EOCRC tumor samples (Fig. 7B). The relative expression of these genes also corroborated with our transcriptomics data analysis as they were identified to be up regulated in the discovery cohort as well (Fig. 7A). These findings suggest that *TNS1* and *MET* were significantly up regulated in EOCRC.

## Discussion

Colorectal cancer is a lethal disease with a gradually increasing risk of occurrence in developing countries. It has also been clearly recorded that there is an increase in frequency of cases of early age onset of colorectal cancer (having no family history) in the past decade. Aetiology of EOCRC is little studied and still obscure. EOCRC with no family history presents a unique type of CRCs that need to be studied in a large context. For this study, we precisely selected 4 tumor samples (median age 43.5 years) having no family history and performed RNA-Seq analysis to identify unique molecular signatures of such tumors. RNA-Seq data of these samples was analyzed using an integrated bioinformatics approach. The analysis was aimed towards the identification of promising DEGs that could be playing a pivotal role during the progression of EOCRC. These DEGs may, in the future, could serve as good biomarkers for prognosis and diagnosis and could be potentially important genes in clinical settings for better understanding of the disease that would help in the decision-making process of clinicians.

Fundamentally big data of transcriptomics faces a severe problem of paired adjacent colonic mucosa. Ideally, the study should comprise of tumor samples paired with normal samples to avoid any biological differences and eliminate the bottleneck factors affecting the selection of the novel candidate biomarker. However, due to the limited number of EOCRC cases, we performed NGS analysis on 4 early age onset CRC and one paired adjacent mucosa. As with other recent studies, we believe, comparing all the tumor sample datasets with one adjacent mucosa would be a good normalizing factor instead of having no adjacent mucosa^10^. Further, to confirm our RNA-Seq data analysis results, gene expression of the identified hub genes was validated in all four tumor samples and their matched normal by QRT-PCR analysis. GO and KEGG pathway analysis were performed on MonaG, iDEP 9.0 and SHINNY GO online servers based on DAVID and STRING database. GEPIA was also used to validate the performance of the screened hub genes in the TCGA database. Finally, total 17 genes were screened from Cytohubba plugin using DMNC and MCC approach. These DEGs were categorically reported in cancer related pathways. These DEGs were then explored on GEPIA server for survival and stage specific differential expression. DEGs such as *CXCL3, IL1B, IL1A, MET* and *TNS1* were found to have significant association with overall survival and *TNS1, CXCL3, MET* and *IL1B* displayed significant association with stage specific differential expression.

Our findings suggest *CXCL3* is significantly expressed in all stages of CRC and is mostly up regulated in all tumor samples. It is a well known biomarker as suggested from previous studies^19^. This protein is a part of the CXC family and involved in T cell trafficking, activation, cellular differentiation and is critical for effector T cell functioning^20^. In the present study, *CXCL3* expression for CRC displayed a significant association with overall survival (*p* < 0.015) (TCGA database), however, Doll et al. (2010) did not find any significant association with overall survival (*p* < 0.10) in a QRT-PCR based study^21^. Similarly, Xiong et al. (2017) reported that patients with elevated *CXCL3* level in CRC tumor tissue had a shorter OS (overall survival) time^22^. In another recent study, up regulated *CXCL3* has been shown to promote cell proliferation, migration by modulating the expression of malignancy related genes in autocrine/paracrine fashion in prostate cancer^23^.

Overexpression of *IL1B* is also well recorded in cancers. *IL1B* is the part of large interleukin family IL1 that contains 11 agonist and antagonist molecules that mediate the regulatory function of inflammatory response^24,25^. The role of *IL1B* in tumor progression has been well established and it is necessary for cellular homeostasis. At the initial stage of cancer progression there are several fine-tuned chemokines and receptors that selectively nurture these balances and protect the normal colonic epithelia. Once this balance is disturbed due to selective pressure, Normal Colorectal Fibroblast (NCF) gets converted to Carcinogen Associated Fibroblast (CAF) and the surrounding tumor microenvironment starts supporting the tumor progression^26^. This concept has selective importance in targeting these cells because CAFs support the tumor cells hiding from the immune surveillance and ease their survival against chemotherapeutic drugs. IL1B along with TGFβ are the most prominent soluble factors inducing NCFs to CAFs and help tumor cells in escaping from chemotherapy^27,28^. It has also been shown to promote EMT in colorectal cancer by modulating *ZEB1*^29^ and in breast cancer treated with doxorubicin. It reduces the antitumor effect of drugs by engaging myeloid regulatory cells resulting in immunosuppression and increased cancer invasiveness^30^.

*MET* or *cMET* was consistently found to be upregulated in our sample groups which was also confirmed by QRT-PCR analysis (Fig. 7). MET works as a mediator of tumor stromal interactor, promoting the angiogenic potential of tumors. Dienstmann et al. (2012) reported that *MET* expression is less in normal colonic mucosa and more in about 59.4% adenomas and tumors^31^. *MET* overexpression was reported in 47.8% adenomas, 66.7% carcinomas and very high (86.4–93.8%) in distant metastasis. Majority of the liver metastasized CRC tumors also have over expressed *MET*^32^. Increased *MET* expression in CRC dysplastic adenoma to advanced carcinoma is indicative of its role in early stages of cancer onset with worse prognosis and high cancer related moralities^33^. *MET*, with its ligand HGF, have been considered to gain metastatic potential and became a critical factor for the origin of cancer^34,35^. Nfonsam et al. (2016) performed the similar expression profiling on the six early age < 50 years and six > 65 years age CRC patients and comparative analysis revealed that MET was uniquely expressed in all under aged tumor samples^11^. On a different note, clinical trial based studies of MET inhibitors (cabozantinib and crizotinib) demonstrated a positive effect on non-small cell lung cancers with least toxicity but in gastrointestinal tumors their efficacy is still questionable and is associated with poor survival prognosis and shorter progression free survival (PFS)^34,36^. These studies suggest the prognostic and therapeutic potential of *MET* gene.

Tensin 1 (*TNS1*) is the member of multidomain transmembrane family located at the focal adhesions, having binding affinity with actin molecules and is involved in various signaling pathways^37^. Tensin contains two domains at its carboxy terminus; PTB (phosphotyrosine binding) and SH2 (Src homology 2) domain. PTB interacts with NPXY motifs of β-actin necessary for perpetuation of β-actin activity and SH2 binds to phosphorylated tyrosine proteins, such as paxillin, Fak, Src, Axl and EGFR. Both the binding activities are necessary for maintenance of cellular homeostasis and β-actin activity is responsible for cell adhesion, proliferation and migration, while other proteins are required for signaling cascade activated by tyrosine kinases^38,39^. *TNS1* was found to be upregulated in our transcriptomic data set of four EOCRC samples (discovery cohort) as well as in the validation cohort of 5 EOCRC samples. However, no other transcriptomic studies are available that describe the *TNS1* expression. *TNS1* expression was downregulated in TCGA database^10^. However, *TNS1* was reported to be significantly upregulated in various tumor tissues as well as cell line-based studies^40,41^. Previous analysis has reported that over expression of *TNS1* is correlated with increased cell migration and metastasis, but the mechanism behind this is still poorly understood. It was reported in cell line-based study that transgelin (TAGLN gene) may regulate the expression of *TNS1* in SW620 cells. Over expressed transgelin induces the *TNS1* expression but vice versa is not true^41^.

Overall our findings suggest that five genes *CXCL3, MET, IL1B, IL1A* and *TNS1* that were screened through the integrated bioinformatics approach have the potential to serve as biomarkers in early age CRC patients. Out of these biomarkers, *TNS1* and *MET* were significantly associated with survival and stage specific expression and were also found to be overexpressed in our validation cohort of early age CRC. Although *TNS1* and *MET* expression was novel for early age criteria based studies and their individual function is also very promising, however, more studies are required to explore their potential as therapeutic or prognostic biomarkers. Finally, this study faces limitation of sample size and older age comparative control. To address this, we selected only early age tumors and compared them with other studies. Still, validation of these genes has to be performed on a larger sample cohort before we can establish them as biomarkers.

## Conclusion

In this study, we performed the integrative bioinformatics approach to identify the novel DEGs unique to early age colorectal cancer patients. This is the first approach to find early age onset biomarkers in Indian CRC patients. We mined genes that are differentially expressed and found that *TNS1* and *MET* are novel for early age onset CRC and may serve as potential biomarkers and novel therapeutic targets for CRC. These biomarkers can also be used in survival and PFS monitoring in CRC.

## Supplementary Information

Supplementary Information.

## Data Availability

The data that supports the findings of this study are available from the corresponding author upon request.

## References

[1] Bray F (2018). Global cancer statistics 2018: GLOBOCAN estimates of incidence and mortality worldwide for 36 cancers in 185 countries. CA Cancer J. Clin..

[2] Siegel RL, Miller KD, Jemal A (2019). Cancer statistics, 2019. CA Cancer J. Clin..

[3] Abualkhair WH (2020). Trends in incidence of early-onset colorectal cancer in the United States among those approaching screening age. JAMA Netw. Open.

[4] Anderson JC, Samadder JN (2018). To screen or not to screen adults 45–49 years of age: That is the question. Am. J. Gastroenterol..

[5] Wolf AMD (2018). Colorectal cancer screening for average-risk adults: 2018 guideline update from the American Cancer Society. CA Cancer J. Clin..

[6] Mathew A (2018). Cancer trends and burden in India. Lancet Oncol..

[7] Dhillon PK (2018). The burden of cancers and their variations across the states of India: The Global Burden of Disease Study 1990–2016. Lancet Oncol..

[8] Mathew A (2019). Colorectal cancer incidence in younger adults in India. Gut.

[9] Mauri G (2019). Early-onset colorectal cancer in young individuals. Mol. Oncol..

[10] Zhu M (2020). Comprehensive RNA sequencing in adenoma-cancer transition identified predictive biomarkers and therapeutic targets of human CRC. Mol. Ther. Nucleic Acids.

[11] Nfonsam V, Xu W, Koblinski J, Jandova J (2016). Gene expression analysis of sporadic early-onset rectal adenocarcinoma. Gastrointest. Cancer (Jersey City).

[12] Ge SX, Son EW, Yao R (2018). iDEP: An integrated web application for differential expression and pathway analysis of RNA-Seq data. BMC Bioinformatics.

[13] Ge SX, Jung D, Yao R (2020). ShinyGO: A graphical gene-set enrichment tool for animals and plants. Bioinformatics.

[14] Xin Z (2020). MonaGO: A novel gene ontology enrichment analysis visualisation system. bioRxiv.

[15] Kanehisa M, Sato Y, Kawashima M, Furumichi M, Tanabe M (2016). KEGG as a reference resource for gene and protein annotation. Nucleic Acids Res..

[16] Szklarczyk D (2019). STRING v11: Protein–protein association networks with increased coverage, supporting functional discovery in genome-wide experimental datasets. Nucleic Acids Res..

[17] Shannon P (2003). Cytoscape: A software environment for integrated models of biomolecular interaction networks. Genome Res..

[18] Tang Z (2017). GEPIA: A web server for cancer and normal gene expression profiling and interactive analyses. Nucleic Acids Res.

[19] Sun G (2019). Identification of differentially expressed genes and biological characteristics of colorectal cancer by integrated bioinformatics analysis. J. Cell. Physiol..

[20] Abron JD (2018). Differential role of CXCR3 in inflammation and colorectal cancer. Oncotarget.

[21] Doll D (2010). Differential expression of the chemokines GRO-2, GRO-3, and interleukin-8 in colon cancer and their impact on metastatic disease and survival. Int. J. Colorectal Dis..

[22] Xiong Y, You W, Wang R, Peng L, Fu Z (2017). Prediction and validation of hub genes associated with colorectal cancer by integrating PPI network and gene expression data. Biomed. Res. Int..

[23] Xin H (2018). Chemokine CXCL3 mediates prostate cancer cells proliferation, migration and gene expression changes in an autocrine/paracrine fashion. Int. Urol. Nephrol..

[24] Borthwick LA (2016). The IL-1 cytokine family and its role in inflammation and fibrosis in the lung. Semin. Immunopathol..

[25] Dinarello CA (2009). Immunological and inflammatory functions of the interleukin-1 family. Annu. Rev. Immunol..

[26] Liu T (2019). Cancer-associated fibroblasts: An emerging target of anti-cancer immunotherapy. J. Hematol. Oncol..

[27] Li M (2016). Targeting of cancer-associated fibroblasts enhances the efficacy of cancer chemotherapy by regulating the tumor microenvironment. Mol. Med. Rep..

[28] Díaz-Maroto NG (2019). Noncanonical TGFβ pathway relieves the blockade of IL1β/TGFβ-mediated crosstalk between tumor and stroma: TGFBR1 and TAK1 inhibition in colorectal cancer. Clin. Cancer Res..

[29] Li Y, Wang L, Pappan L, Galliher-Beckley A, Shi J (2012). IL-1β promotes stemness and invasiveness of colon cancer cells through Zeb1 activation. Mol. Cancer.

[30] Zitvogel L, Kepp O, Galluzzi L, Kroemer G (2012). Inflammasomes in carcinogenesis and anticancer immune responses. Nat. Immunol..

[31] Dienstmann R (2012). Molecular profiling of patients with colorectal cancer and matched targeted therapy in phase I clinical trials. Mol. Cancer Ther..

[32] Gayyed MF, Abd El-Maqsoud NMR, El-Hameed El-Heeny AA, Mohammed MF (2015). c-MET expression in colorectal adenomas and primary carcinomas with its corresponding metastases. J. Gastrointest. Oncol..

[33] Zeng Z-S (2008). c-met gene amplification is associated with advanced stage colorectal cancer and its liver metastases. Cancer Lett..

[34] Mo H-N, Liu P (2017). Targeting MET in cancer therapy. Chronic Dis. Transl. Med..

[35] Osada S (2010). Effect of hepatocyte growth factor on progression of liver metastasis in colorectal cancer. Hepatogastroenterology.

[36] Lee SJ (2018). c-MET overexpression in colorectal cancer: A poor prognostic factor for survival. Clin. Colorectal Cancer.

[37] Lo SH (2017). Tensins. Curr. Biol..

[38] Georgiadou M (2017). AMPK negatively regulates tensin-dependent integrin activity. J. Cell. Biol..

[39] Muharram G (2014). Tensin-4-dependent MET stabilization is essential for survival and proliferation in carcinoma cells. Dev. Cell.

[40] Burghel GJ (2013). Identification of candidate driver genes in common focal chromosomal aberrations of microsatellite stable colorectal cancer. PLoS ONE.

[41] Zhou H (2017). Elevated transgelin/TNS1 expression is a potential biomarker in human colorectal cancer. Oncotarget.

