# Supplementary material for: Transcriptomic landscape of early age onset of colorectal cancer identifies novel genes and pathways in Indian CRC patients
Source: Sci Rep. 2021 Jun 3;11:11765. doi: 10.1038/s41598-021-91154-x (PMC8175339; doi:10.1038/s41598-021-91154-x)
Supplement: Supplementary file 1 — Supplementary Information. [file 41598_2021_91154_MOESM1_ESM.docx]

**Title page**

**Title:** Transcriptomic landscape of early age onset of colorectal cancer identifies novel genes and pathways in Indian CRC patients

**Authors:** Manish Pratap Singh^1^, Sandhya Rai^1^, Nand K Singh^1^, Sameer Srivastava*^1^

^1^ Department of Biotechnology Motilal Nehru National Institute of Technology Allahabad, Prayagraj (India)

**Corresponding Author:**

Dr. Sameer Srivastava

Department of Biotechnology

Motilal Nehru National Institute of Technology Allahabad,

Prayagraj, India 211004

Email:sameers@mnnit.ac.in

Supplementary Figure 1: Basic alignment statistics of RNA seq data.

Supplementary Figure 2: Expression heat map of common 95 DEGs in all samples.

Supplementary Figure 3: Gene expression profile of most significant 17 mutual hub genes with matched normal on the TCGA database using COAD (colon adenocarcinoma) cohort.

Supplementary Table 1. Transcriptomic data summary of all samples.

Supplementary Table 2. Number of significant differentially expressed transcripts between adjacent mucosa and tumor samples.

Supplementary Table 3. List of primers used in the validation of differentially expressed genes among tumor samples.

Supplementary Figure 1:


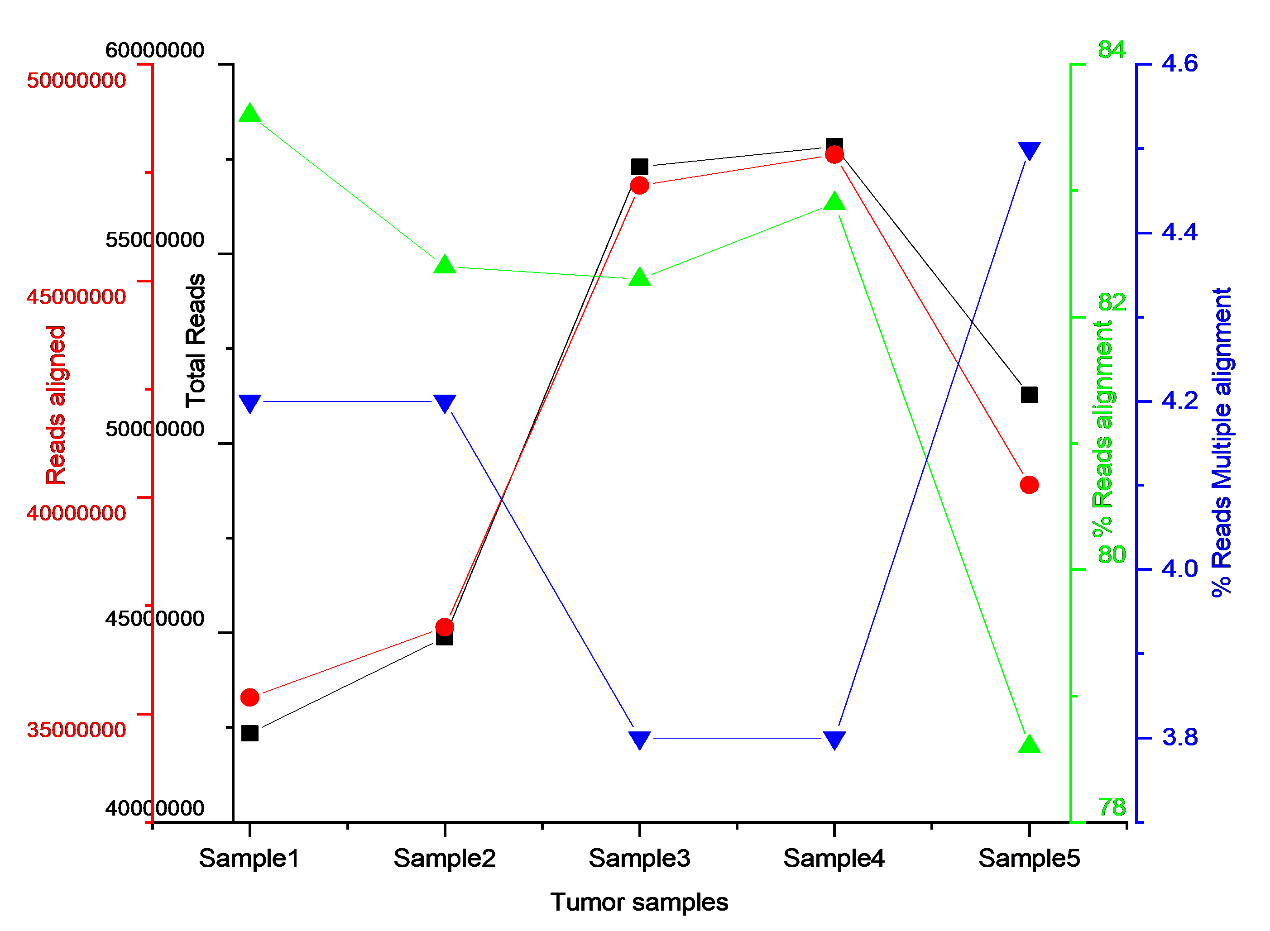


Supplementary Figure 2:


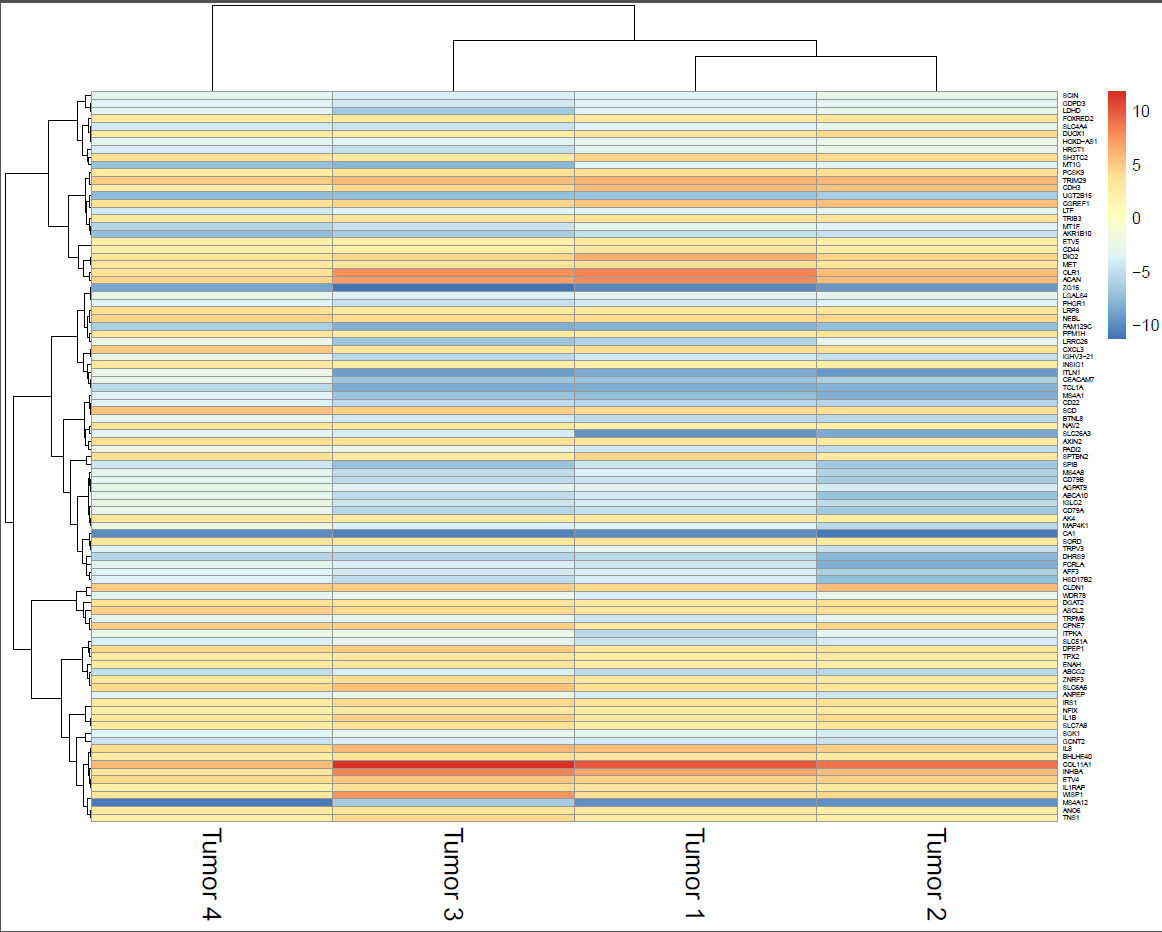


Supplementary Figure 3:


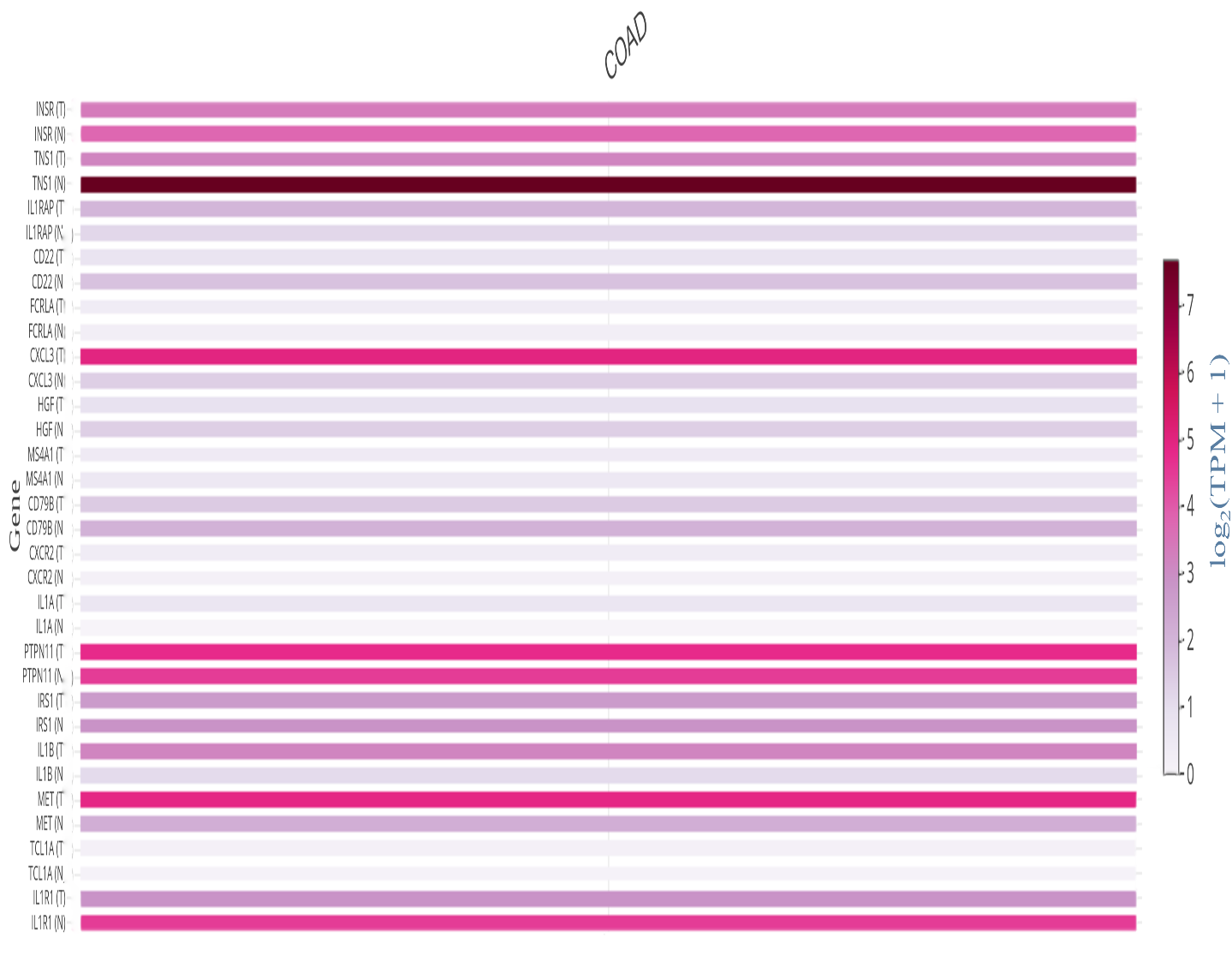


Supplementary Table 1. Transcriptomic data summary

| Sample_ID | Raw_Data_GB | Processed_Data_GB |
| --- | --- | --- |
| Sample1 | 6.6 | 6.3 |
| Sample2 | 7.0 | 6.6 |
| Sample3 | 8.9 | 8.4 |
| Sample4 | 9.0 | 8.5 |
| Sample5 | 8.1 | 7.5 |

Supplementary Table 2. Number of significant differentially expressed transcripts between adjacent mucosa and tumor samples.

| **Sample5 vs Sample1** | | | | |
| --- | --- | --- | --- | --- |
| **Category** | **Total** | **UP** | **DOWN** | **NEUTRAL** |
| **Both Adjacent mucosa and Tumor samples** | 15903 | 4131 | 2256 | 9516 |
| **Only Adjacent mucosa** | 381 | NA | NA | NA |
| **Only Tumor** | 313 | NA | NA | NA |
| **Sample5 vs Sample2** | | | | |
| **Category** | **Total** | **UP** | **DOWN** | **NEUTRAL** |
| **Both Adjacent mucosa and Tumor samples** | 15951 | 3834 | 2366 | 9751 |
| **Only Adjacent mucosa** | 435 | NA | NA | NA |
| **Only Tumor** | 388 | NA | NA | NA |
| **Sample5 vs Sample3** | | | | |
| **Category** | **Total** | **UP** | **DOWN** | **NEUTRAL** |
| **Both Adjacent mucosa and Tumor samples** | 16261 | 4685 | 2694 | 8882 |
| **Only Adjacent mucosa** | 443 | NA | NA | NA |
| **Only Tumor** | 359 | NA | NA | NA |
| **Sample5 vs Sample4** | | | | |
| **Category** | **Total** | **UP** | **DOWN** | **NEUTRAL** |
| **Both Adjacent mucosa and Tumor samples** | 16057 | 3958 | 2383 | 9716 |
| **Only Adjacent mucosa** | 332 | NA | NA | NA |
| **Only Tumor** | 331 | NA | NA | NA |

*Transcripts having Log2FoldChange >=1 for UP and <=-1 for DOWN has been considered as significant DEGs.

Supplementary Table 3. List of primers used in the QRT-PCR analysis for validation of differentially expressed genes among tumor samples.

|  | **Gene** | **Sequence 5’-3’** |
| --- | --- | --- |
|  | COL11A1 F | ACTTCTGACTGCCTCTGCTCG |
|  | COL11A1 R | TGGACGCACAACCATCATACA |
|  | OLR1 F | ACTGGAGGGACAGATCTCAGC |
|  | OLR1 R | TGGTGGTGAAGTTCCATTTGCT |
|  | ACAN F | ATTTCATCGACCCCATGCACC |
|  | ACAN R | CTGATAGGCACTGTTGACCCG |
|  | INHBA F | TCTCCACATACCCGTTCTCCC |
|  | INHBA R | AGGCCGTCAAGAAGCACATTT |
|  | DIO2 F | CCCGTAAGCTATGTTGGCGTT |
|  | DIO2 R | CATCAGATGGCTGGGCGATAC |
|  | SPTBN2 F | GCATTTGCCTCTCGAGTCAGTT |
|  | SPTBN2 R | ACCACTTAGAGTGCACGGAGAC |
|  | ANO6 F | TCCTCAGCCTTTGGTACACTCA |
|  | ANO6 R | GGTGGCTGGATTGAAGAAAGCA |
|  | ZG16 F | CAACGGAGACCTGGAGGAGAT |
|  | ZG16 R | GGACGGCATTGAAACTTGTG |
|  | CA1 F | TAGTGTCTCCTACAACCCAGCC |
|  | CA1 R | GCTGTCAGAGAAAGGACCACCT |
|  | MS4A12 F | CATACCCAACCCTTACCCACC |
|  | MS4A12 R | AGATTCCCGGAGATGTGATGC |
|  | ITLN1 F | GGACCTGTTCTTCGTCTCCATC |
|  | ITLN1 R | GGTCATGTCACAGAAGGTCTGG |
|  | TCL1A F | GGTATCGTCCATCAGGGTAGAG |
|  | TCL1A R | TACAGTTACGGGTGCTCTTGC |
|  | LRRC26 F | GACGCGGTTGTGGTCCAGCAG |
|  | LRRC26 R | GCTGTCGCCTTGGCCTGTCTG |
|  | FAM129C F | GGGACCGCTCGAGTCGTG |
|  | FAM129C R | GACAGTCGGTCCATGCCTTG |
|  | **MET F** | **TGCACAGTTGGTCCTGCCATGA** |
|  | **MET R** | **CAGCCATAGGACCGTATTTCGG** |
|  | **TNS1 F** | **ACTCCAGAGGAGGAGCCATTGA** |
|  | **TNS1 R** | **TGTGGCTTCTGGAGACTGGTTC** |
|  | GAPDH F | GGATTTGGTCGTATTGGG |
|  | GAPDH R | GGAAGATGGTGATGGGATT |
